# A New Medical Library Building

**Published:** 1900-06

**Authors:** 


### A NEW MEDICAL LIBRARY BUILDING.

THE dedication of the new library building of the Medical Society of the County of Kings at 1313 Bedford Avenue, Brooklyn, N.Y., took place at 2 p.m., Saturday, May 19, 1900, under the following order of exercises:

In the chair, Lewis S. Pilcher, President Medical Society of the County of Kings; invocation, Rev. A J. Lyman, D.D.; address, George M. Gould, Philadelphia, President of the Association of Medical Librarians; address, James R. Chadwick, Boston, Librarian, Boston Medical Library; address, Abraham Jacobi, New York, Chairman Board of Trustees, New York Academy of Medicine; Transferring of the Building to the Board of Trustees, William Maddren, Chairman of the Building Committee; Acceptance of the Building by the Trustees and Transferring the same to the Society, Frank E. West, Chairman of the Board of Trustees; Acceptance of the Building by the Society, Lewis S. Pilcher, President of the Society; Benediction, Rev. John P. Chidwick, U.S.N. A reception was given to the ladies from 5 to 6 p.m.

The JOURNAL is pleased to acknowledge the receipt of an invitation to attend, and regrets that it could not accept.

This society has done itself great credit in building a home and in establishing a library therein. It is an example that ought to be imitated in Buffalo and for the sake of stimulating such a movement we present a brief history of the Kings County Medical Society's building:

MOVEMENT BEGUN IN 1891.

The start toward getting the beautiful new home for the Medical Society of the County of Kings was made in 1891, when Dr. Joseph Hunt, the librarian of the society, said that the library "already demands more room." This library consists of more than 40,000 volumes, many of which are exceedingly valuable. The old, small and cramped quarters made it necessary to store a considerable number of these volumes. Following Dr. Hunt's report, the desire for more commodious and central quarters grew stronger each year. The society was not a rich one, however, and the thought of going into a scheme which would make necessary the expenditure of considerable money was to most of the members, who are at all times the busiest of men, a rather arduous proceeding.

The initiative was taken by Dr. McNaughton, when in his annual address as president in 1894, he emphasised the growing need and made the recommendation that some action be taken at once. A committee was appointed that was known as the "Jumbo Committee," to distinguish it from the minor committees, and it consisted of two members from each of the wards of the city, and two members from each of the county towns. It was organised in September of that year, with Dr. F. H. Stuart as treasurer and Dr. Myerle as secretary. Through the persistent efforts of this committee $17,000 was raised in pledges.

The most serious problem next to the financial one, which had to be settled, was that of a site. After considerable discussion between those who desired a location in the neighborhood of Fulton Street and Flatbush Avenue and those who favored the Bedford district of the city, the latter element won, largely, it was said by its opponents, through the inability to find a suitable property at a reasonable price in the downtown neighorhood. In the spring of 1897, the president of the society, the chairman of the "Jumbo Committee" and the chairman of the Board of Trustees, recommended the site in Bedford Avenue, opposite the 23d Regiment Armory. This selection met the approval of the members of the society, as the extension of the Kings

County Elevated Railroad, and various other improvements in transportation throughout that district made it as convenient of access as any part of the city. The amount paid for the lot was $17,500, one-half in cash.

THE CHOOSING OF THE PLANS.

A final building committee of five was appointed to consider thoroughly all the details, adopt plans for a structure and have general supervision of the work until its completion. It was composed of Drs. Frank E. West, George McNaughton, Wm. Maddren, Charles Jewett and William Browning, each representing some leading interest in the society. Frank Freeman was appointed consulting architect, and the plans of Wald & Cranford were chosen from twenty designs.

The building occupies a rectangle 59 by 85 feet. It is colonial in design, and executed in brick with stone trimmings. It is a three story and basement fire-proof structure, with special provisions for the safety of the library. In the basement a place has been reserved for bicycles, and the remaining space is devoted to a heating and ventilating apparatus and the elevator machinery. Coming into the building from Bedford Avenue, one steps into a vestibule in what is to be known as the main entrance hall. This is in the nature of a foyer for the auditorium, entrance to which is gained through three doorways. Around the foyer are a reception parlor, a women's room and a cloakroom.

At the right is the main office, the desk of which commands a view of all entrances. The auditorium will seat nearly 400, and is to be lighted chiefly from skylights extending across the building and over the speaker's platform. On the second story is the reading room, which occupies the entire front of the building. Here are to be fire-places, tables, current journal cases, card catalogue, cabinet for photographs and rare books. A feature of the library is the alcoves for private study. Adjacent to the reading room is a conversation room. The rear of the second floor is devoted to the stockroom, which has a capacity of about 100,000 volumes. Everything here is absolutely fireproof. The third story is devoted to the apartments for the librarian and custodian of the building, storage rooms and two large section rooms, which may be used separately or in conjunction.

In transferring the building to the Board of Trustees, Dr. William Maddren, chairman of the building committee, referred to the history of the procurement of the new building. For several years there had been an increasing realisation of the inadequacy of facilities for library work. Most of the members remembered the pathetic piling up in cellars, closets and all over the floor of valuable books and precious material. This involved not only the impossibility of making a proper use of them, but also their rapid deterioration and destruction, as well as the continued risk of loss at the hands of collectors. This also dampened the ardor of any would-be givers.

Dr. Maddren continued as follows:

Most of those present can remember only too well the cramped meeting quarters in old Bridge Street home. The president had to suspend the session if a noisy vehicle passed along; the hygienic sense of the editor of *The Medical Journal* was outraged by the condition of the atmosphere when meetings of any size occurred; the oldest member was afraid his life might be shortened by the collapse of the building; the inhabitants of the house were frightened nearly out of their wits by the frequent creakings of the timbers at night. Finally, the city officials stepped in and put a stop to any further crowding of the building, and directed our beloved trustees to shore up the sinking walls. The outlook became more and more discouraging, and no one seemed able and inclined to come to the relief of our society —and by that is meant practically the whole profession of Kings county.

This society is indebted to its former president, Dr. George McNaughton, for the initiatory act in the procurement and erection of this building. The building committee also feels that his name should receive the place of honor for his enthusiasm, zeal and earnest effort in the realisation and adaptation of this building for the uses of this society. In 1894, at the April meeting of this society, the president, Dr. McNaughton, delivered an inaugural address, in which he dwelt upon the inadequacy and unfitness of our place of meeting and the need of a suitable and fireproof structure for our library. Prompt action was urged upon the society, and a committee was appointed to consider the recommendations of the president.

The total cost of our undertaking for building and site will reach early $90,000, and we are assured at the present time it will cost a large increase on the amount paid to duplicate the building. To meet this outlay our funds have come from subscriptions, mostly from members, from the sale of our former property, and from the building loan. And at a time when our need of help was perhaps the greatest the wives and families of many members of the society organised themselves into a "Woman's Auxiliary to the Committee on New Building," and to raise money to help along our new building gave, at great expense of time and effort, the now famous and successful Græco-Roman Festival. The sum received from this source amounted to $17,130.10, and the committee will ever bear in grateful remembrance the Woman's Auxiliary for this large and timely help.

